# Implementation of an AI-Based Clinical Decision Support System Predicting In-Hospital Cardiac Arrest in General Wards: A Multicenter Staggered-Implementation Study in Secondary Hospitals in Korea

**DOI:** 10.3390/diagnostics16111682

**Published:** 2026-05-29

**Authors:** Minjeong Kim, Dongjoon Yoo, Eunbi Noh, Yongwook Jeong, Minsoo Kim, Kyung-Jae Cho, Mincheol Kim, You Dong Sohn, Gyu Chong Cho

**Affiliations:** 1 Division of Pulmonology, Department of Internal Medicine, Shihwa Medical Center, Siheung 15063, Republic of Korea; aube1800@naver.com; 2 Department of Critical Care Medicine and Emergency Medicine, Inha University Hospital, Incheon 22332, Republic of Korea; djinyoo@gmail.com; 3 VUNO Inc., Seoul 06164, Republic of Korea; eunbi.noh@vuno.co (E.N.); minsoo.kim@vuno.co (M.K.); kcho035@vuno.co (K.-J.C.); mincheol.kim@vuno.co (M.K.); 4 Department of Emergency Medicine, Naeun Hospital, Incheon 21565, Republic of Korea; tomorrow0810@gmail.com; 5 Department of Emergency Medicine, School of Medicine, Hallym University, Seoul 05355, Republic of Korea

**Keywords:** artificial intelligence, clinical deterioration, deep learning, DeepCARS, early warning score, in-hospital cardiac arrest, real-world evidence, secondary care, resource-constrained settings, low resource hospital, patient outcome assessment

## Abstract

**Background/Objectives:** In-hospital cardiac arrest (IHCA) remains a devastating event associated with high morbidity and mortality among general ward patients. While Rapid Response Systems (RRS) can help identify deteriorating patients, maintaining these systems in secondary hospitals is frequently hindered by severe fiscal and personnel constraints. Consequently, evidence regarding the real-world clinical effectiveness of artificial intelligence software as a medical device (AI-SaMD) for predicting deterioration in such resource-constrained settings remains limited. **Methods:** We conducted a retrospective analysis on a multicenter, staggered-implementation study evaluating 164,761 eligible adult general ward admissions across three secondary hospitals in South Korea. The intervention involved deploying an AI-SaMD (DeepCARS), which utilizes four routine vital signs to predict ward IHCA within 24 h. The primary outcome was ward IHCA. Secondary outcomes included in-hospital mortality and length of stay (LOS). Exploratory analyses investigated the mechanisms of clinical outcomes by evaluating lead-times to interventions, outcomes in sepsis subgroups, changes in care directives, and post-arrest neurological outcomes. **Results:** AI-SaMD implementation was associated with a 21% reduction in ward IHCA incidence (adjusted rate ratio 0.79; 95% CI, 0.65–0.96; *p* = 0.016) and a 15% reduction in in-hospital mortality (aRR 0.85; 95% CI, 0.79–0.90; *p* < 0.001), alongside significantly shorter hospital and intensive care unit LOS. These associations were also observed in patients with sepsis (IHCA aRR 0.71; 95% CI, 0.54–0.93; *p* = 0.013). Lead-times to critical care intervention and to antibiotic escalation were numerically shorter in the AI-SaMD group by 16.3 h (*p* = 0.066) and 2.6 h (*p* = 0.523); poor neurological outcome at discharge among ward IHCA cases was 85/108 (78.7%) in the AI-SaMD group versus 63/102 (61.8%) in the standard-care group (aRR 1.13; 95% CI, 0.99–1.33; *p* = 0.058); and the full-code death rate did not differ between groups (aRR 0.94; 95% CI, 0.76–1.15)—none of these additional analyses reached statistical significance. **Conclusions:** In secondary hospitals unable to operate an RRS due to fiscal limitations, implementation of an AI-SaMD as an additional informational layer was associated with lower ward IHCA and in-hospital mortality. The AI-SaMD may serve as an actionable and scalable additional safety layer for general-ward patients in resource-constrained environments where RRS infrastructure is not feasible. Although this was a multicenter, large-scale study, the present analysis was retrospective and quasi-experimental in design; rigorous randomized studies are needed to confirm these associations.

## 1. Introduction

In-hospital cardiac arrest (IHCA) remains a frequent and devastating event among hospitalized patients. In the United States alone, approximately 292,000 patients experience IHCA annually [1]. Globally, the reported incidence in adults ranges from 1.2 to 10 cases per 1000 admissions [2]. Although survival to hospital discharge has improved over time, long-term prognosis remains poor; a systematic review and meta-analysis estimated 1-year survival after IHCA at 13.4% [3].

Hospital safety initiatives once focused on the “efferent limb” (post-arrest response) but now emphasize the “afferent limb,” using Early Warning Scores (EWS) and/or Rapid Response Systems (RRSs) to detect deterioration earlier [4]. Despite a Cochrane review showing no consistent patient-centered benefit of combined EWS and RRS implementation [5], American Heart Association (AHA) and Society of Critical Care Medicine (SCCM) guidelines still recommend structured early warning and hospital-wide surveillance in the general ward [6,7].

These inconsistent effects may partly reflect limitations of conventional track-and-trigger scores such as National Early Warning Score (NEWS) and NEWS2, which generate excessive false alerts while still missing true deterioration [8,9]. AI has shown substantial promise for early detection of inpatient clinical deterioration, with AI-based EWSs outperforming conventional scores [10]. However, retrospective comparisons based only on discrimination metrics such as the Area Under the Receiver Operating Characteristic curve (AUROC) guarantees only feasibility rather than clinical utility; the DECIDE-AI guideline therefore classifies such evidence as phase 0–1 and highlights the gap between algorithmic accuracy and real-world effectiveness [11]. Notably, even a high-performing AI system (AUROC > 0.85) failed to improve patient-centered outcome in a large, randomized trial versus usual care [12]. This discrepancy may reflect implementation challenges and underscores that strong discrimination does not necessarily translate into better clinical outcomes [13].

Recent policy and academic discourse, including JAMA publications and AHA statements, has emphasized evaluating AI according to its intended clinical use and real-world effects on patient-centered outcomes [13,14]. Yet prospective outcome-driven evidence remains limited [4,13,14].

To date, three large-scale studies have evaluated advanced EWSs with patient-centered endpoints. Although all reported improved outcomes, their findings should be interpreted in context: Escobar et al. involved substantial increases in RRS staffing and operational restructuring [4,15]; Winslow et al. also restructured the RRS and modified protocols [4,16]; and Park et al. evaluated implementation in a center with an existing RRS [17].

However, most evidence for advanced EWS models comes from tertiary or well-resourced hospitals with mature RRS, whereas many secondary hospitals cannot sustain such systems. In the United States, maintaining an RRS has been estimated to cost more than US$1 million over 5 years in a medium-sized hospital [18]. In Japan, only 2% of small hospitals maintained an RRS, compared with 20% of large hospitals [19]. Although South Korea has promoted RRS nationally, phase 3, launched on 1 April 2025, included only 55 hospitals [20]. In smaller hospitals, implementation remains limited mainly by fiscal constraints and shortages of trained personnel [18,19,21,22,23], as highlighted during the recent Korean healthcare crisis [24].

The AI-based Software as a Medical Device (AI-SaMD) used in this study, VUNO Med^®^-DeepCARS (version 1.3.2), estimates ward IHCA risk within the next 24 h using only 4 classic and routine vital signs as input. It obtained Korean, European, and partial U.S. regulatory approval, and showed clinical effectiveness in a recent large, controlled trial with a 35% reduction in mortality [17]. However, its real-world effect in secondary hospitals without an RRS remains unclear. In light of this evidence gap, we hypothesized that implementation of an AI-SaMD without additional staffing or mandated response protocols would be associated with improved patient outcomes in low-resource hospital environments.

## 2. Materials and Methods

### 2.1. Study Design, Reporting, and Ethics

We conducted a retrospective analysis on a multicenter staggered-implementation study to evaluate whether implementation of an AI-SaMD for ward IHCA risk monitoring was associated with improved outcomes in hospitals without an RRS or additional staffing. This study followed the STROBE statement and DECIDE-AI guideline [11,25] (Supplementary File S2 and S3).

The institutional review boards (IRB) of each participating hospital approved the study (KANGDONG IRB 2025-10-004; SHMC_IRB_2025_P010; NH_IRB_250912-P001) and waived informed consent because only routine clinical data were used for a retrospective analysis. All procedures were conducted in accordance with the Declaration of Helsinki [26].

### 2.2. Study Setting and Population

We included all adults aged 19 years or older admitted to general wards during the study period. We excluded patients younger than 19 years, those with a documented Physician Orders for Life-Sustaining Treatment (POLST) indicating non-resuscitation status or care directives [27], admissions spanning the implementation date, and ICU-only admissions without any general-ward stay.

### 2.3. Intervention

Before implementation, all general-ward healthcare professionals (HCPs) received structured training on score interpretation, the clinical implications of elevated scores, and recommended responses based on prior studies [17,28,29,30,31,32]. No hospital-wide mandatory response protocol was introduced, and the AI-SaMD system was implemented as an additional informational decision-support layer within routine workflows. The alert threshold was set at 90 at all hospitals, consistent with prior studies and approximately corresponding to NEWS of 5 or higher [28,29,30,31,32].

### 2.4. Pre-Implementation and Post-Implementation Periods

Before implementation, none of the hospitals operated an RRS or MET. Conventional EWSs were available in the EMR, but varied across hospitals (NEWS, modified early warning score [MEWS], or single-parameter track-and-trigger systems [SPTTS]) and were not linked to a standardized escalation protocol. Clinical responses therefore remained at clinician discretion. For pre-post comparison, AI-SaMD scores were retrospectively calculated, based on prior studies [15,16].

After implementation, the AI-SaMD system was integrated into the EMR vital-sign interface alongside existing EWSs and automatically generated a risk score whenever any of the four vital signs was recorded. Alerts were displayed to bedside nurses and responsible physicians when the score was 90 or higher, without mandatory notification. This threshold was selected to align with high-severity escalation triggers used in NEWS-based frameworks [33,34].

### 2.5. Study Period and Staggered Implementation

**Figure 1:**
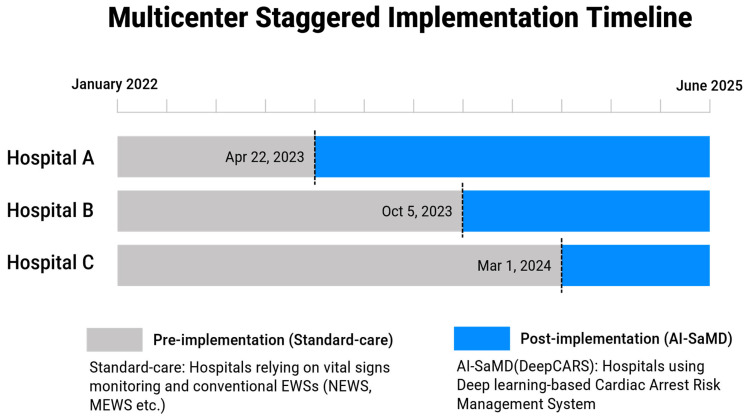
Study Design.

Admissions after each hospital’s implementation date were assigned to the AI-SaMD group, whereas admissions before implementation were assigned to the standard-care group. Patients whose admissions spanned the implementation date and transition period were excluded from the main analysis to eliminate the risk of treatment contamination resulting from overlapping exposures. Across sites, the pre- and post-implementation periods comprised 63 and 63 hospital-months, respectively (Figure 1).

### 2.6. Study Outcomes

For neurologic outcome analysis, CPC scores were dichotomized as good neurologic outcome (CPC 1–2) and poor neurologic outcome (CPC 3–5) [35]. Additional secondary analyses examined ward IHCA and in-hospital mortality among patients with sepsis.

### 2.7. Data Collection and Preprocessing

Data were extracted from hospital EMRs, including demographic characteristics, admission and discharge data, admitting department, diagnoses, comorbidities, surgical status, ICU utilization, CPR records, in-hospital mortality, and end-of-life documentation (care directives). Illness severity at admission was assessed using the Sequential Organ Failure Assessment (SOFA) score based on the first available measurements during the index hospitalization [36,37]. Detailed definitions, per-component data sources, and missingness handling are provided in Supplementary File S1.

When laboratory values required for SOFA calculation were unavailable on the admission date, the most recent value within 30 days before admission was carried forward, consistent with established preoperative-assessment practice in non-ICU settings as endorsed by the European Society of Anaesthesiology and Intensive Care [38]. Components remaining missing after this step were assumed to indicate normal organ function (0 points), following the convention established in the Sepsis-3 consensus framework and explicitly retained in the recent SOFA-2 update [39,40]. Per-component missingness rates, before and after the 30-day pre-admission LOCF window, are reported in Supplementary Table S4.

Comorbidity burden was assessed using the Charlson Comorbidity Index (CCI) derived from ICD-10 codes and documented medical history [41]. Hypertension was included as an additional comorbidity variable. Time-stamped vital-sign data, risk scores, and AI-SaMD alerts timestamps were extracted for all admissions.

### 2.8. Statistical Analysis

All eligible adult general-ward admissions during the predefined study period at each participating hospital were included.

The primary unit of analysis was individual hospital admissions. Continuous variables were summarized as mean (standard deviation), and categorical variables as frequency (%). Baseline characteristics were compared using Student’s *t*-test or chi-square test, as appropriate.

Primary and secondary outcomes—ward IHCA, in-hospital mortality, and poor neurologic outcome at discharge—were analyzed using multivariable Poisson generalized linear mixed-effects models (GLMMs) with a log link and hospital as a random intercept to estimate adjusted rate ratios (aRRs). Covariates included age, sex, CCI, hypertension, SOFA score at admission, season of admission, and surgical department status. Calendar year was not included as a covariate in the primary outcome model because the standard-care and AI-SaMD groups co-existed only during a brief overlap period (2023 to Feb 2024). Secular-trends were instead addressed indirectly in a sensitivity analysis using propensity score matching with exact matching on hospital and calendar year (Supplementary Table S5).

Total hospital and ICU LOS were summarized descriptively as median (interquartile range) and analyzed using inverse probability of censoring weighting (IPCW)-weighted Gamma GLMMs with an identity link and hospital as a random intercept, adjusted for the same covariates as in the primary models. IPCW was applied because in-hospital death may truncate observed LOS and introduce informative censoring bias [42,43]. The period coefficient was interpreted as the adjusted mean difference in LOS between the post- and pre-implementation periods within the same hospital. ICU LOS analysis was restricted to admissions with any ICU stay.

All tests were two-sided, and *p* values < 0.05 were considered statistically significant. Analyses were performed using R version 4.5.2 and Python version 3.13.1.

### 2.9. Secondary Analysis

In additional analysis, we examined ward IHCA and in-hospital mortality among patients with sepsis, according to the Centers for Disease Control and Prevention (CDC) Adult Sepsis Event surveillance criteria [44]. Detailed operational definitions are provided in Supplementary File S1.

Lead-time analyses evaluated the time from the first AI-SaMD alert to critical care intervention and antibiotic escalation among admissions with at least one qualifying alert and a recorded response. In the standard-care group, alert timing was retrospectively derived from calculated AI-SaMD score, consistent with prior landmark AI-EWS studies [15,16]. Outcomes were analyzed using Gamma GLMMs with an identity link, hospital as a random intercept, and the same covariates as in the primary analysis.

Department-stratified analyses assessed heterogeneity across specialties for ward IHCA and in-hospital mortality (Supplementary Table S2). Admissions were classified into nine department categories; surgical departments were dichotomized into essential and minor categories using the Lancet Commission on Global Surgery framework [45], ensuring that the subgroup analysis accurately reflected differing degrees of clinical severity.

To evaluate whether AI-SaMD alerts prompted timely care directives discussions and reduced potentially futile resuscitations, we examined the ‘full-code death’ rate, defined as deaths following CPR while maintaining full-code status, as a process measure of end-of-life care transitions, following Escobar et al. [15] (Supplementary Table S3).

### 2.10. Sensitivity Analysis

To evaluate the robustness of the primary findings, we performed several sensitivity analyses. Admissions ongoing at the hospital-specific implementation date were excluded in the main analysis, but in sensitivity analyses were reassigned to the pre-implementation period, and an additional 3.5-day washout window was applied (Supplementary Table S1). Adjusted risk differences (aRDs) on the absolute scale were additionally estimated by the same Poisson GLMMs to verify consistency on the additive scale (Supplementary Table S6). We also performed one-to-one nearest-neighbor PSM (caliper, 0.2 SD of the logit) with exact matching on hospital and calendar year, and assessed unmeasured confounding using the E-value sensitivity analysis [46] (Supplementary Table S5).

## 3. Results

### 3.1. Study Population

**Figure 2:**
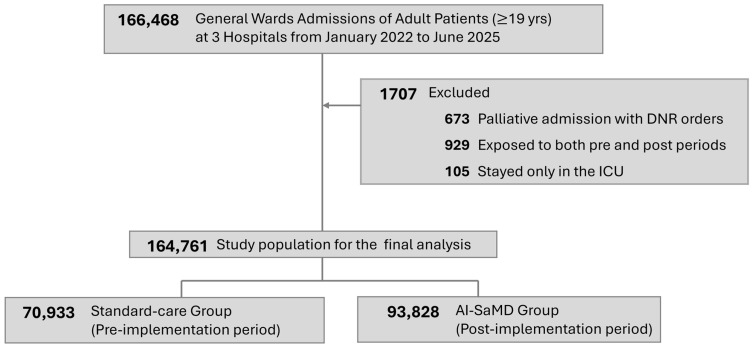
Flow Diagram of Study Participants.

A total of 166,468 adult general ward admissions from three hospitals between January 2022 and June 2025 were identified. After excluding 1707 admissions—palliative DNR status (*n* = 673), exposure to both pre- and post-implementation periods (*n* = 929), or ICU-only stays (*n* = 105)—164,761 admissions were included in the final analysis: 70,933 in the standard-care group and 93,828 in the AI-SaMD group (Figure 2).

### 3.2. Baseline Characteristics

Table 1 summarizes the baseline characteristics of the study population. Although sex distribution was similar between groups, the AI-SaMD group was slightly older and had a higher burden of comorbidity and illness severity than the standard-care group, including a higher prevalence of hypertension, higher mean SOFA scores, higher mean CCI values, and a greater proportion of sepsis. In addition, the distributions of department type, admission department, hospital, and season of admission also differed between the two groups.

### 3.3. Primary and Secondary Outcomes

Ward IHCA was significantly lower in the AI-SaMD group (aRR 0.79; 95% CI, 0.65–0.96; *p* = 0.016) (Table 2). In-hospital mortality was also significantly lower in the AI-SaMD group (aRR 0.85; 95% CI, 0.79–0.90; *p* < 0.001). Total hospital LOS and ICU LOS were significantly shorter in the AI-SaMD group than in the standard-care group (aMD −0.51 days; 95% CI, −0.61 to −0.42; *p* < 0.001; and aMD −1.32 days; 95% CI, −1.84 to −0.80; *p* < 0.001, respectively). Among ward IHCA cases, poor neurological outcome at discharge occurred in 85 of 108 (78.7%) in the AI-SaMD group and 63 of 102 (61.8%) in the standard-care group; this difference did not reach statistical significance (aRR 1.13; 95% CI, 0.99–1.33; *p* = 0.058). As this analysis is restricted to patients who experienced ward IHCA, it should be interpreted as a within–IHCA-cohort comparison rather than a population-level estimate.

### 3.4. Sepsis Subgroup Analysis

Among 8336 admissions with sepsis, ward IHCA occurred in 51 of 3066 admissions (1.66%) in the standard-care group and 56 of 5270 admissions (1.06%) in the AI-SaMD group, while in-hospital mortality occurred in 301 (9.82%) and 351 (6.66%), respectively (Table 2). The AI-SaMD group had significantly lower risks of ward IHCA (aRR 0.71; 95% CI, 0.54–0.93; *p* = 0.013) and in-hospital mortality (aRR 0.78; 95% CI, 0.69–0.87; *p* < 0.001) than the standard-care group.

### 3.5. Lead-Time to Clinical Response

Among 2579 admissions with AI-SaMD alerts, lead-times to critical care intervention and to antibiotic escalation were numerically shorter in the AI-SaMD group by 16.3 h (aMD −0.68 days; 95% CI, −1.39 to 0.04; *p* = 0.066) and 2.6 h (aMD −0.11 days; 95% CI, −0.44 to 0.22; *p* = 0.523), respectively, but neither difference reached statistical significance (Table 2).

### 3.6. Department-Stratified and Care Directive Analyses

In department-stratified analyses, a significant reduction in ward IHCA was observed only in cardiology (aRR, 0.70; 95% CI, 0.50–0.99). Significant reductions in in-hospital mortality were observed in pulmonology (aRR, 0.62; 95% CI, 0.55–0.70) and essential surgical specialties (aRR, 0.76; 95% CI, 0.57–0.99), whereas most other estimates were below 1 but not statistically significant (Supplementary Table S2).

Among patients without prior care directives (patients with full-code), AI-SaMD implementation was not associated with a significant difference in full-code death among admissions with full-code status at baseline (aRR, 0.94; 95% CI, 0.76–1.15).

### 3.7. Sensitivity Analysis

The associations between AI-SaMD implementation and improved outcomes were consistent across alternative transition-period definitions. ARRs ranged from 0.78 to 0.80 for ward IHCA and from 0.84 to 0.85 for in-hospital mortality. In the PSM analyses with exact matching on hospital and calendar year, the AI-SaMD group showed a significantly lower risk of in-hospital mortality (RR, 0.71; 95% CI, 0.57–0.89) and a directionally consistent but non-significant reduction in ward IHCA (RR, 0.73; 95% CI, 0.40–1.32) compared with the standard-care group (Supplementary Table S5). The E-value for the mortality association was 2.17 (lower confidence limit, 1.51).

## 4. Discussion

In this multicenter, staggered-implementation study of 166,468 general ward admissions across three secondary hospitals in South Korea, the implementation of DeepCARS as an additional informational layer for patient safety was associated with lower ward IHCA incidence, reduced in-hospital mortality, and shorter hospital and ICU LOS. The reductions in ward IHCA incidence and in-hospital mortality were 21% and 15%, respectively, and these findings remained consistent across various sensitivity analyses (PSM and transition-period alterations). To our knowledge, this is the first large-scale evaluation of patient-centered outcomes demonstrating the clinical effectiveness of an AI-SaMD in secondary hospitals without RRS and without the infusion of additional resources.

### 4.1. Novelty in the Context of Previous Studies

Previous large-scale studies of AI-based EWSs have reported improved patient-centered outcomes [15,16,17]. However, those studies were predominantly conducted in resource-rich settings or hospitals with established RRS infrastructure, and evidence remains limited for secondary hospitals without RRS. Our study design aligns with a recent randomized trial that implemented a commercial AI-SaMD using a passive visual display of AI risk trajectories without a mandated response protocol, which failed to improve patient outcomes [47]. The relatively low predictive performance (C-statistic: 0.63 to 0.73) reported in its retrospective development [48], the confounding effects of the COVID-19 pandemic, and the specific implementation strategy utilized may have influenced those negative results.

In our study, the AI-SaMD was implemented in three hospitals where an RRS or MET could not be established due to fiscal constraints. Prior to implementation, all HCPs received comprehensive training on the system, including explanations of the relationship between high-risk scores and fatal outcomes, as demonstrated in a previous multicenter prospective study [31]. The risk score was displayed alongside routine vital signs in the EMR for bedside nurses and attending physicians, with the expectation that it would prompt early reassessment and intervention. Although implemented in a resource-constrained setting without a dedicated efferent limb, the intervention was associated with lower rates of ward IHCA and in-hospital mortality.

The implementation of the AI-SaMD was also associated with shorter hospital and ICU LOS. These findings are consistent with the earlier recognition and timely treatment of clinical deterioration, which may support faster recovery and a more efficient redistribution of scarce healthcare resources, such as ICU beds.

Unlike the recent non-randomized controlled trial of the same AI-SaMD by Park et al. [17], which was conducted in a center with established RRS infrastructure, the present study provides large-scale, multicenter real-world evidence in a complementary and underrepresented setting—secondary hospitals without an RRS or additional staffing—rather than a clean causal demonstration of effectiveness, which the retrospective, quasi-experimental design cannot support. Generalizability beyond the three Korean secondary hospitals studied here remains to be confirmed in prospective, ideally randomized or cluster-randomized studies.

### 4.2. Exploratory Analysis of Outcome Results

This exploratory analysis was designed to evaluate the underlying mechanisms driving the patient-centered outcomes associated with AI-SaMD implementation. We focused on the real-world actionability and behavioral changes among HCPs. This approach contrasts with commonly used post hoc mathematical interpretability methods (e.g., SHAP, LIME) intended to address the “black-box” nature of AI models; such mathematical explanations often fail to translate into tangible clinical actions that improve patient outcomes and are frequently perceived by HCPs as unhelpful [49,50,51]. Because timely intervention is crucial for improved outcomes such as mortality in critical clinical deterioration events like sepsis, we quantitatively evaluated the time to intervention. Furthermore, in an era where patient self-determination regarding life-sustaining treatments is paramount, we analyzed changes in care directives [15]. We also conducted extensive adjustments for confounding factors, subgroup analyses, and sensitivity analyses to rigorously test for potential biases inherent in the retrospective analysis.

### 4.3. Lead-Time Findings and Their Implications

Among the selected subgroup of alert-positive admissions, the post-implementation group showed numerically shorter times from the first alert to both critical care intervention and antibiotic escalation, although neither difference reached statistical significance. These findings should therefore be interpreted as hypothesis-generating rather than as evidence of a specific causal mechanism. They are consistent with—but do not prove—the possibility that AI-SaMD supported earlier recognition and response, which would be in line with current guidelines for the management of sepsis and clinical deterioration outside the ICU [7,52]. Because alert acknowledgment, HCP fidelity, and compliance were not directly measured, and because the analysis was restricted to admissions in which an alert occurred, this interpretation should be approached with caution.

### 4.4. Findings in Patients with Sepsis

The improvement in outcomes tended to be greater in patients with sepsis compared to the overall population. This finding is clinically plausible given the highly time-sensitive nature of sepsis and the well-established association between delayed antimicrobial treatment and adverse outcomes [52,53]. Although the time to antibiotic escalation was 2.6 h shorter in the AI-SaMD group, this difference lacked statistical significance; therefore, this interpretation requires caution. The trend toward a shorter time to antibiotic administration may suggest earlier recognition and appropriate treatment of impending organ failure. Further studies are necessary to clarify whether the primary benefit of the AI-SaMD is concentrated within the sepsis subpopulation or reflects a mixed effect across all deteriorating patients.

### 4.5. Care Directives and DNR Changes

An initial hypothesis was that the observed reduction in ward IHCA may have been influenced also by proactive end-of-life care recommendations, leading to a higher rate of reclassification to non-resuscitation status. While respecting patient autonomy and acknowledging current RRS guidelines that encourage the appropriate redistribution of intensive care resources through DNR directives, the full-code death analysis (Supplementary Table S3) is consistent with, rather than supportive of, this artifactual explanation. Among patients without a prior care directive at admission, the adjusted rate ratio for death following CPR was similar between cohorts. These findings are therefore consistent with clinical prevention of ward IHCA rather than with administrative differences in care directives, although the quasi-experimental design does not allow this distinction to be established with certainty.

### 4.6. Neurological Outcomes After IHCA

Although poor neurological outcomes at discharge did not differ significantly between groups, the point estimate was above 1 in the AI-SaMD group, contrasting with the improvements seen in the main outcomes. One hypothesis is that the AI-SaMD implementation may have prevented more reversible deterioration events from progressing to ward IHCA, leaving a residual cohort of IHCA patients with potentially greater severity and poorer neurological prognoses. Prior studies of refractory IHCA populations have reported similar patterns of poor neurological outcomes [1,2,3].

### 4.7. Department-Level Heterogeneity

The department-stratified analyses were heterogeneous across clinical specialties. A significant reduction in ward IHCA was observed in cardiology, whereas significant reductions in in-hospital mortality were observed in pulmonology and essential surgical specialties; most other departments demonstrated trends toward improvement without reaching statistical significance. These differences may reflect variations in the pathophysiological pathways linking clinical deterioration to cardiac arrest or death across specialties. In cardiology, preventing ward IHCA may not necessarily translate into lower mortality, as outcomes are often strongly influenced by the irreversible severity of the underlying cardiac disease. In contrast, in respiratory and surgical departments, earlier recognition may be more likely to successfully interrupt pathways leading to fatal outcomes. Given the exploratory nature of these analyses and the limited number of events in several strata, these findings should be interpreted cautiously.

### 4.8. Findings from Sensitivity Analysis

The primary findings were robust across multiple sensitivity analyses. Effect estimates remained directionally consistent under alternative transition-period definitions and an extended 3.5-day washout window (Supplementary Table S1), in PSM analyses with exact matching on hospital and calendar year (Supplementary Table S5), and when expressed as adjusted risk differences on the absolute scale (Supplementary Table S6).

### 4.9. Limitations

This study has several limitations. First, given the retrospective nature of this analysis, observed associations should not be interpreted as definitive evidence of causality. Second, the AI-SaMD group had a greater baseline illness burden; although addressed via multivariable adjustment and PSM, residual confounding from unmeasured acuity may persist. Third, because the temporal window during which standard-care and AI-SaMD groups co-existed was narrow (2023 to February 2024), secular calendar-time trends could not be directly adjusted in the primary outcome model; we addressed this indirectly through PSM with exact matching on hospital and calendar year. The 2024 Korean national healthcare disruption is the most salient concrete instance of this broader concern, as it overlapped almost entirely with the post-implementation period at Hospital C. Although the AI-SaMD group showed consistently better outcomes across three hospitals with staggered implementation timings—a pattern that would be difficult to explain by a single nationwide event alone—residual confounding from this and other unmeasured secular changes cannot be fully excluded, and the observed associations should accordingly be interpreted as supportive rather than confirmatory. Fourth, because lead-time analyses in the standard-care group relied on retrospectively calculated AI-SaMD scores rather than real-time alerts, the true lead-time advantage may have been underestimated. Fifth, in the lead-time analysis, time zero was defined as the first AI-SaMD alert during hospitalization. Because no standardized definition of time zero exists—even within the well-studied context of sepsis [53]—our lead-time estimates should be interpreted cautiously and may not be directly comparable across studies using alternative definitions. Sixth, deep learning-based AI models can show substantial performance variability across datasets and training settings. Even with identical inputs, prediction horizons, target outcomes, and training methodologies, variations in the training data can lead to significantly different model behaviors and performance [54,55,56]. Consequently, these findings should not be extrapolated to other AI-based EWS models without dedicated validation. Seventh, this study was prespecified as an implementation outcomes evaluation; the algorithmic performance of the AI-SaMD has been extensively characterized in dedicated prior studies [31,32]. Alert-level workflow data fell outside the scope of the present evaluation. Eighth, because the AI-SaMD was implemented as a lightweight informational safety layer without a mandated response protocol, direct human–AI interaction (clinician adherence and alert acknowledgement) was not collected—a limitation also noted in milestone evaluations of AI-based early warning systems [15,16,57]. Finally, because the study was conducted in three secondary hospitals in South Korea, the generalizability of these findings to other healthcare systems requires further validation.

### 4.10. Future Directions

These findings support the feasibility and potential clinical benefit of implementing AI-SaMD in resource-constrained secondary hospitals. Future prospective randomized or cluster-randomized studies are needed to establish causality and clarify the mechanism of benefit through direct measurement of clinician responses to alerts. Further study of sepsis-specific pathways may help refine understanding of the population-level impact of selective IHCA prevention.

## 5. Conclusions

In secondary hospitals without RRS, implementation of AI-SaMD as an additional informational layer for patient safety was associated with reduced ward IHCA incidence, in-hospital mortality, and hospital LOS. The pattern of findings observed in the full-code death and lead-time analyses may be compatible with upstream prevention of clinical deterioration, but the quasi-experimental design does not allow this mechanism to be established. These results support a potential role for AI-SaMD as an additional, scalable safety layer for general-ward patients in resource-constrained settings where established RRS infrastructure is not feasible. The present study does not compare AI-SaMD with a fully implemented RRS, and the findings should not be interpreted as implying that AI-SaMD substitutes for RRS where RRS is available. Rigorous prospective randomized or cluster-randomized studies are needed before causal or policy-level conclusions can be drawn.

## Figures and Tables

**Table 1:** Baseline Characteristics of Study Participants.

Characteristic		Total (*N* = 164,761)	AI-SaMD (*n* = 93,828)	Standard-Care (*n* = 70,933)
**Sex**				
	Male	81,580 (49.5)	46,327 (49.4)	35,253 (49.7)
	Female	83,181 (50.5)	47,501 (50.6)	35,680 (50.3)
**Age, y**				
		60.6 ± 17.1	60.7 ± 16.9	60.4 ± 17.3
**Hypertension**				
	Yes	66,060 (40.1)	38,317 (40.8)	27,743 (39.1)
	No	98,701 (59.9)	55,511 (59.2)	43,190 (60.9)
**SOFA score at admission**				
		0.82 ± 1.30	0.87 ± 1.32	0.76 ± 1.27
**CCI**				
		1.18 ± 1.70	1.25 ± 1.75	1.10 ± 1.64
**Department type**				
	Surgical	80,405 (48.8)	44,105 (47.0)	36,300 (51.2)
	Non-surgical	84,356 (51.2)	49,723 (53.0)	34,633 (48.8)
**Admission department**				
	Essential surgery	50,221 (30.5)	27,125 (28.9)	23,096 (32.6)
	Pulmonology	25,943 (15.7)	15,054 (16.0)	10,889 (15.4)
	Cardiology	24,255 (14.7)	14,512 (15.5)	9743 (13.7)
	Neurology	21,394 (13.0)	11,782 (12.6)	9612 (13.6)
	Gastroenterology	13,878 (8.4)	9023 (9.6)	4855 (6.8)
	Nephrology	13,745 (8.3)	7535 (8.0)	6210 (8.8)
	Oncology	8121 (4.9)	4938 (5.3)	3183 (4.5)
	Other	4455 (2.7)	2428 (2.6)	2027 (2.9)
	Minor surgery	2749 (1.7)	1431 (1.5)	1318 (1.9)
**Hospital**				
	A	63,473 (38.5)	32,374 (34.5)	31,099 (43.8)
	B	39,201 (23.8)	20,374 (21.7)	18,827 (26.5)
	C	62,087 (37.7)	41,080 (43.8)	21,007 (29.6)
**Season of admission**				
	Spring	46,956 (28.5)	26,596 (28.4)	20,360 (28.7)
	Summer	41,438 (25.1)	21,851 (23.3)	19,587 (27.6)
	Autumn	34,076 (20.7)	20,975 (22.4)	13,101 (18.5)
	Winter	42,291 (25.7)	24,406 (26.0)	17,885 (25.2)
**Sepsis**				
	Yes	8336 (5.1)	5270 (5.6)	3066 (4.3)
	No	156,425 (94.9)	88,558 (94.4)	67,867 (95.7)
**AI-SaMD Alarm**				
	Yes	2579 (1.6)	1240 (1.3)	1339 (1.9)
	No	162,182 (98.4)	92,588 (98.7)	69,594 (98.1)

Data are presented as *n* (%) for categorical variables and mean ± SD for continuous variables. **Abbreviations:** CCI, Charlson Comorbidity Index; SD, standard deviation; SOFA, Sequential Organ Failure Assessment.

**Table 2:** Association Between AI-SaMD Implementation and Clinical Outcomes.

		AI-SaMD Group	Standard-Care Group	Adjusted Estimate (95% CI)	*p* Value
**All included**		***n*** **= 93,828**	***n*** **= 70,933**		
	Ward IHCA, no. (%)	108 (0.12%)	102 (0.14%)	0.79 (0.65, 0.96)	0.016
	In-hospital mortality, no. (%)	983 (1.05%)	897 (1.26%)	0.85 (0.79, 0.90)	<0.001
	Length of stay, days ^†^	3.90 (6.19)	3.95 (6.15)	−0.51 (−0.61, −0.42)	<0.001
	ICU length of stay, days ^†^	3.29 (8.81)	3.67 (8.34)	−1.32 (−1.84, −0.80)	<0.001
	Poor neurological outcome at discharge, no. (%) *	85 (78.70%)	63 (61.76%)	1.13 (0.99, 1.33)	0.058
**Sepsis cohort**		***n*** **= 52** **70**	***n*** **= 306** **6**		
	Ward IHCA, no. (%)	56 (1.06%)	51 (1.66%)	0.71 (0.54, 0.93)	0.013
	In-hospital mortality, no. (%)	351 (6.66%)	301 (9.82%)	0.78 (0.69, 0.87)	<0.001
**AI-SaMD a** **lert** **cohort**		***n*** **= 1240**	***n*** **= 1339**		
	Alert to critical care intervention, days ^†^	0.69 (3.40)	0.77 (3.80)	−0.68 (−1.39, 0.04)	0.066
	Alert to antibiotic escalation, days ^†^	1.82 (4.86)	1.90 (5.24)	−0.11 (−0.44, 0.22)	0.523

^†^ Length of stay and alert-response times as median (IQR), where IQR denotes the interquartile range calculated as Q3–Q1. Ward IHCA and in-hospital mortality are shown as count (%); * CPC at discharge was analyzed only among patients with ward IHCA. Adjusted rate ratios (aRRs) and 95% CIs were estimated using Poisson generalized linear mixed models with a hospital-level random intercept. Adjusted mean differences (aMDs) and 95% CIs were estimated using gamma generalized linear mixed models with a hospital-level random intercept. Length-of-stay models used inverse probability of censoring weighting for in-hospital death. All models were adjusted for sex, age, Charlson Comorbidity Index, hypertension, SOFA score at admission, season of admission, and department type. **Abbreviations**: aMD, adjusted mean difference; aRR, adjusted rate ratio; CI, confidence interval;ICU, intensive care unit; IHCA, in-hospital cardiac arrest; IQR, interquartile range; SOFA, Sequential Organ Failure Assessment.

## Data Availability

The institutional datasets used in this study, along with de-identified results, are available upon reasonable request for purposes such as systematic review or meta-analysis, with approval from the corresponding authors and the IRBs.

## Supplementary Materials

The following supporting information can be downloaded at: https://www.mdpi.com/article/10.3390/diagnostics16111682/s1. References [11,36,37,44] are cited in the supplementary materials. Supplementary File S1: Supplementary Methods; Supplementary File S2: STROBE checklist; Supplementary File S3: DECIDE-AI checklist; Table S1: Sensitivity Analyses of Ward IHCA and In-Hospital Mortality According to Transition-Period Definition; Table S2: Department-Stratified Analysis of Ward IHCA and In-Hospital Mortality; Table S3: Adjusted Rate Ratios for Full-Code Death Among Patients Without Prior Care Directives at Admission; Table S4:Per-Component Missing Rate of Baseline SOFA Score Under Two Laboratory Inclusion Windows; Table S5: Propensity Score Matched Analysis (Exact-Matched on Hospital and Calendar Year) of Ward IHCA and In-Hospital Mortality with E-value Sensitivity Analysis; Table S6: Association Between AI-SaMD Implementation and Clinical Outcomes.

## Abbreviations

AHA	American Heart Association
AI	artificial intelligence
AI-SaMD	artificial intelligence software as a medical device
AUROC	area under the receiver operating characteristic curve
CCI	Charlson Comorbidity Index
CDC	Centers for Disease Control and Prevention
CPC	Cerebral Performance Category
CPR	cardiopulmonary resuscitation
DECIDE-AI	Developmental and Exploratory Clinical Investigation of Decision Support Systems Driven by Artificial Intelligence
DNR	do-not-resuscitate
EMR	electronic medical record
EWS	early warning system
GLMM	generalized linear mixed model
HCP	healthcare professional
ICD-10	International Classification of Diseases, Tenth Revision
ICU	intensive care unit
IHCA	in-hospital cardiac arrest
IPCW	inverse probability of censoring weighting
IRB	institutional review board
LOS	length of stay
MET	medical emergency team
MEWS	Modified Early Warning Score
NEWS	National Early Warning Score
NEWS2	National Early Warning Score 2
POLST	Physician Orders for Life-Sustaining Treatment
PSM	propensity score matching
RRS	rapid response system
SCCM	Society of Critical Care Medicine
SOFA	Sequential Organ Failure Assessment
SPTTS	single-parameter track-and-trigger system
STROBE	Strengthening the Reporting of Observational Studies in Epidemiology
