# Supplementary material for: Implementation of an AI-Based Clinical Decision Support System Predicting In-Hospital Cardiac Arrest in General Wards: A Multicenter Staggered-Implementation Study in Secondary Hospitals in Korea
Source: Diagnostics (Basel). 2026 May 29;16(11):1682. doi: 10.3390/diagnostics16111682 (PMC13257196; doi:10.3390/diagnostics16111682)
Supplement: Supplementary file 1 [file diagnostics-16-01682-s001.zip › Supplementary FileS1_Supplementary Methods.pdf]

Supplementary FileS1_Supplementary Methods

Supplementary Methods 1. Detailed description of SOFA score at admission

Supplementary Methods 2. Operational definition of sepsis based on ASE criteria

Supplementary Methods 1. Detailed description of SOFA score at admission

The Sequential Organ Failure Assessment (SOFA) score was originally developed to describe the degree of organ dysfunction in critically ill patients by evaluating six organ systems: respiratory, cardiovascular, hepatic, coagulation, renal, and neurological. Following the Third International Consensus Definitions for Sepsis and Septic Shock (Sepsis-3) [36], the score has become the gold standard for defining sepsis, where an acute increase of 2 points or more represents life-threatening organ dysfunction **[Box 1]**.

Challenges in Non-ICU settings

In this study, admission severity was evaluated by calculating the SOFA score based on clinical data available at the time of hospitalization. However, since the original SOFA framework was designed for intensive care unit (ICU) settings, several variables required for SOFA scoring are not routinely measured or consistently recorded in general ward or non-ICU settings. These include variable such as the PaO_2/FiO_2 ratio, mean arterial pressure, and the Glasgow Coma Scale (GCS). In addition, detailed information on vasopressor administration, such as dosage and titration over time required for cardiovascular SOFA scoring, is not routinely documented in general ward settings.

Adapted Framework for General Wards

To address these limitations, we utilized an adapted SOFA framework optimized for non-ICU data availability, following the methodology proposed by Raymond et al. [37]. In this approach, the cardiovascular and respiratory components were modified to use more readily available clinical measures,

- Cardiovascular: Systolic blood pressure (SBP) was used instead of MAP or vasopressor dosing.
- Respiratory: Peripheral oxygen saturation (SpO_2) and oxygen therapy status were used in place of PaO_2/FiO_2.

The remaining four components—central nervous system (CNS), renal (creatinine), hepatic

(bilirubin), and hematologic (platelets)—remained consistent with the original SOFA scoring thresholds. However, the urine output criterion within the renal component was excluded as it is not routinely monitored in general wards.

Operationalization and Scoring

For the CNS component, we utilized the AVPU scale (Alert, Verbal, Pain, Unresponsive) instead of the Glasgow Coma Scale (GCS), as GCS is infrequently recorded in general ward settings. A score of 0 points was assigned for an 'Alert' status (AVPU A or GCS 15), while 1 point was assigned for any other state (AVPU V, P, or U). For patients with end-stage renal disease (ESRD), the creatinine component was assigned the maximum of 4 points.

The total admission SOFA was calculated by summing the scores of all six organ system components. Consistent with the original SOFA concept, points were assigned only for new or worsening abnormalities relative to the patient's baseline.

The Raymond SOFA scoring system is summarized in **[Box 2]**, and the final study-specific operationalization is presented in **[Box 3]**. For laboratory variables required for SOFA calculation, if measurements were unavailable on the admission date, the most recent value within 30 days prior to admission was carried forward using a last-observation-carried-forward (LOCF) approach. Remaining missing component values were imputed using component-specific median values within the study cohort.

Box 1. SOFA score

	Cardiovascular	Respiratory	CNS	Creatinine	Platelets	Bilirubin
Score	Mean arterial pressure (mmHg)	PaO_2/FiO_2	GCS	mg/dL	$\times 10^9$/L	mg/dL
0	MAP ≥70	≥400	15	<1.2	≥150	<1.2
1	MAP <70	<400	13-14	1.2-1.9	<150	1.2-1.9
2	Dopamine <5 or dobutamine (any dose)	<300	10-12	2.0-3.4	<100	2.0-5.9

3	Dopamine 5.1-15 or epinephrine ≤0.1 or norepinephrine ≤0.1	<200 with respiratory support	6-9	3.5-4.9	<50	6.0-11.9
4	Dopamine >15 or epinephrine >0.1 or norepinephrine >0.1	<100 with respiratory support	<6	≥5.0	<20	>12.0

PaO2, Partial pressure of oxygen; FiO2, Fraction of inspired oxygen; CNS, central nervous system; GCS, Glasgow Coma Score

Box 2. Modified SOFA (mSOFA) scoring system proposed by Raymond et al.

	Cardiovascular	Respiratory	CNS	Creatinine	Platelets	Bilirubin
Score	Systolic BP (mmHg)	Oxygen Saturation (%)	GCS	µmol/L	× 10^9/L	µmol/L
0	BP ≥100	≥90% pre-O_2	15	<110	≥150	<20
1	BP <100, pre-IV fluids	<90% pre- O_2 therapies	13-14	110-170	<150	20-32
2	BP <100, post-IV fluids	<90% despite O_2 therapies	10-12	171-299	<100	33-101
3	Inotropes	Ventilated	6-9	300-400	<50	102-204
4			<6	>400	<20	>204

CNS, central nervous system; GCS, Glasgow Coma Score

Box 3. Study-specific operationalization of the modified SOFA (mSOFA) components

	Cardiovascular	Respiratory	CNS	Creatinine	Platelets	Bilirubin
Score	Systolic BP (mmHg)	Oxygen Saturation (%)	AVPU	mg/dL	× 10^9/L	mg/dL
0	BP ≥100	≥90% pre-O_2	A	<1.2	≥150	<1.2
1	BP <100, pre-IV fluids	<90% pre- O_2 therapies	V/P/U	1.2-1.9	<150	1.2-1.9
2	BP <100, post-IV fluids	<90% despite O_2 therapies		2.0-3.4	<100	2.0-5.9
3	Inotropes	Ventilated		3.5-4.9	<50	6.0-11.9
4				≥5.0	<20	>12.0

CNS, central nervous system; GCS, Glasgow Coma Score

Supplementary Methods 2. Operational definition of sepsis based on ASE criteria

In general ward settings, laboratory measurements required for SOFA-based sepsis definitions are not routinely obtained on a daily basis, resulting in substantial missingness. Therefore, for the sepsis subgroup analysis, sepsis was defined using an electronic health record-based framework derived from the Centers for Disease Control and Prevention (CDC) Adult Sepsis Event (ASE) surveillance definition [44]. Sepsis was not defined using the Sepsis-3 framework because Sepsis-3 provides a conceptual definition intended for clinical diagnosis rather than a standardized operational definition that can be consistently implemented for automated identification using routinely collected electronic health data. In this study, sepsis events were identified strictly according to the CDC ASE surveillance criteria, and the study-specific operational definition is detailed below.

Adult Sepsis Event (ASE) framework

The ASE definition identifies sepsis events using two components: presumed infection and organ dysfunction, enabling standardized surveillance using routinely collected EHR data.

Presumed infection was defined as the initiation of ≥4 qualifying antimicrobial days (QAD) starting within a time window extending from 2 calendar days before to 2 calendar days after the date of blood culture collection. A qualifying antimicrobial day refers to any day on which systemic antimicrobial therapy is administered. A new antimicrobial was defined as an antimicrobial agent not administered within the preceding 2 calendar days.

Organ dysfunction was defined as the occurrence of at least one of the following events within the same time window surrounding blood culture collection:

- Initiation of a new vasopressor infusion (norepinephrine, dopamine, epinephrine, phenylephrine, or vasopressin)
- Initiation of invasive mechanical ventilation
- Doubling of serum creatinine or decrease by ≥50% of estimated glomerular filtration rate (eGFR) relative to baseline, excluding patients with end-stage renal disease

- Total bilirubin ≥2.0 mg/dL and increase by 100% from baseline
- Platelet count <100 × 10^9/L and ≥50% decline from baseline
- Serum lactate ≥2.0 mmol/L

The ASE framework provides separate definitions of baseline organ function for community-onset and hospital-onset events. In this study, baseline organ function was defined using the most favorable values observed during hospitalization, consistent with the community-onset event definition.

The ASE onset date was defined as the earliest day within the ± 2-day window surrounding blood culture collection on which any of the following occurred: blood culture collection, initiation of qualifying antimicrobial therapy (first QAD), or fulfillment of organ dysfunction criteria. As illustrated in the figure, HD #1 was identified as the sepsis onset date because it was the earliest qualifying event among the first QAD (HD #1), blood culture collection (HD #2), and initiation of invasive mechanical ventilation (HD #4).

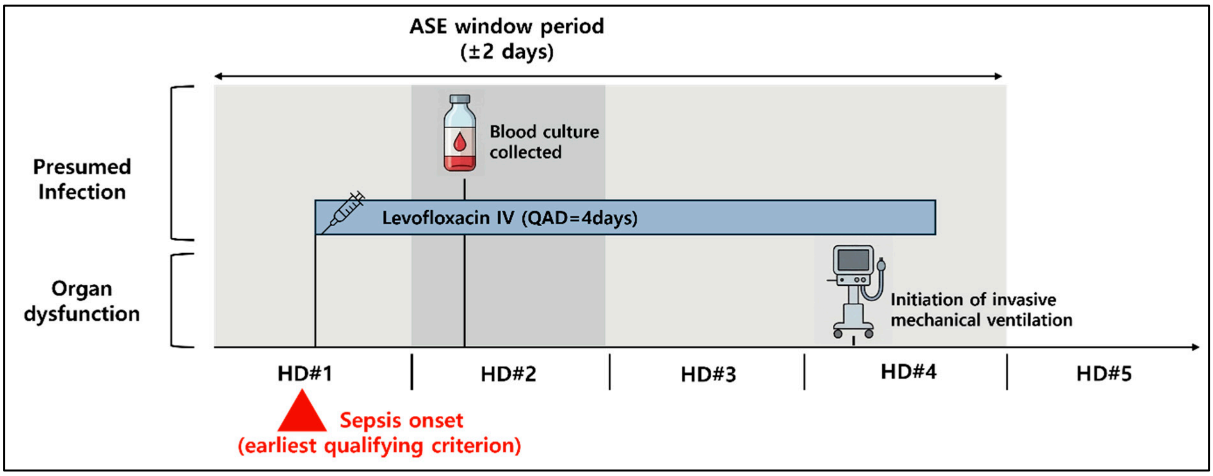

Missing values for ASE criteria were imputed as absence of the corresponding criterion (0). Forward filling of laboratory or physiologic variables was not applied.

These simplified criteria approximate organ dysfunction captured by the SOFA score while enabling reliable automated extraction from EHR.
