# Supplementary material for: Implementation of an AI-Based Clinical Decision Support System Predicting In-Hospital Cardiac Arrest in General Wards: A Multicenter Staggered-Implementation Study in Secondary Hospitals in Korea
Source: Diagnostics (Basel). 2026 May 29;16(11):1682. doi: 10.3390/diagnostics16111682 (PMC13257196; doi:10.3390/diagnostics16111682)
Supplement: Supplementary file 1 [file diagnostics-16-01682-s001.zip › Supplementary Table final.pdf]

Supplementary Table S1. Sensitivity Analyses of Ward IHCA and In-Hospital Mortality According to Transition-Period Definition

Transition-period definition	Outcome	Event / N (%)		Adjusted RR (95% CI)
		AI-SaMD group, no. /N (%)	Standard-care group, no. /N (%)	
**No exclusion**	Ward IHCA	109 / 93,903 (0.12%)	107 / 71,930 (0.15%)	0.78 (0.65–0.94)
	In-hospital mortality	992 / 93,903 (1.06%)	940 / 71,930 (1.31%)	0.84 (0.79–0.89)
**Main analysis**	Ward IHCA	108 / 93,828 (0.12%)	102 / 70,933 (0.14%)	0.79 (0.65–0.96)
	In-hospital mortality	983 / 93,828 (1.05%)	897 / 70,933 (1.26%)	0.85 (0.79–0.90)
**±3.5 days**	Ward IHCA	109 / 93,451 (0.12%)	102 / 70,649 (0.14%)	0.80 (0.66–0.96)
	In-hospital mortality	989 / 93,451 (1.06%)	903 / 70,649 (1.28%)	0.85 (0.79–0.90)

Adjusted rate ratios (aRRs) and 95% CIs were estimated using Poisson generalized linear mixed models with a hospital-level random intercept. All models were adjusted for sex, age, Charlson Comorbidity Index, hypertension, SOFA score at admission, season of admission, and department type. Main analysis excluded admissions within the prespecified transition period around AI-SaMD implementation; alternative analyses used no exclusion or a ±3.5-day transition window.

Abbreviations: aRR, adjusted rate ratio; CI, confidence interval; IHCA, in-hospital cardiac arrest; SOFA, Sequential Organ Failure Assessment.

Supplementary Table S2. Department-Stratified Analysis of Ward IHCA and In-Hospital Mortality

A. Ward IHCA

Department	N	Event (%)		Adjusted RR (95% CI)
		AI-SaMD group, no. /N (%)	Standard-care group, no. /N (%)	
Surgical				
Essential surgery[a]	50,221	46 (0.17%)	57 (0.25%)	0.76 (0.57–0.99)
Minor surgery[b]	2,749	0 (0.00%)	0 (0.00%)	—
Non-surgical				
Pulmonology	25,943	214 (1.42%)	290 (2.66%)	0.62 (0.55–0.70)
Cardiology	24,255	395 (2.72%)	291 (2.99%)	1.00 (0.90–1.12)
Gastroenterology	13,878	110 (1.22%)	78 (1.61%)	0.83 (0.67–1.03)
Neurology	21,394	69 (0.59%)	57 (0.59%)	0.97 (0.75–1.24)
Oncology	8,121	78 (1.58%)	65 (2.04%)	0.82 (0.65–1.05)
Nephrology	13,745	59 (0.78%)	53 (0.85%)	0.96 (0.73–1.25)
Other[c]	4,455	12 (0.49%)	6 (0.30%)	1.20 (0.59–2.43)

B. In-hospital Mortality

Department	N	Event (%)		Adjusted RR (95% CI)
		AI-SaMD group, no. /N (%)	Standard-care group, no. /N (%)	
Surgical				
Essential surgery[a]	50,221	46 (0.17%)	57 (0.25%)	0.76 (0.57–0.99)
Minor surgery[b]	2,749	0 (0.00%)	0 (0.00%)	—
Non-surgical				
Pulmonology	25,943	214 (1.42%)	290 (2.66%)	0.62 (0.55–0.70)
Cardiology	24,255	395 (2.72%)	291 (2.99%)	1.00 (0.90–1.12)
Gastroenterology	13,878	110 (1.22%)	78 (1.61%)	0.83 (0.67–1.03)
Neurology	21,394	69 (0.59%)	57 (0.59%)	0.97 (0.75–1.24)
Oncology	8,121	78 (1.58%)	65 (2.04%)	0.82 (0.65–1.05)
Nephrology	13,745	59 (0.78%)	53 (0.85%)	0.96 (0.73–1.25)
Other[c]	4,455	12 (0.49%)	6 (0.30%)	1.20 (0.59–2.43)

N denotes the total number of admissions included in each department stratum across both cohorts. Adjusted rate ratios (aRRs) and 95% CIs were estimated using Poisson generalized linear mixed models with a hospital-level random intercept, adjusted for sex, age, Charlson Comorbidity Index, hypertension, SOFA score at admission, and season of admission.

[a] Essential surgery includes general surgery, cardiothoracic surgery, obstetrics/gynecology, and orthopedic surgery. [b] Minor surgery includes plastic surgery, ophthalmology, and interventional procedures. [c] Other includes emergency medicine, nuclear medicine, dermatology, and rehabilitation medicine. [d] — indicates not estimable because of zero events in one or both cohorts or model non-convergence.

Abbreviations: aRR, adjusted rate ratio; CI, confidence interval; IHCA, in-hospital cardiac arrest; SOFA, Sequential Organ Failure Assessment.

Supplementary Table S3. Adjusted Rate Ratios for Full-Code Death Among Patients Without Prior Care Directives at Admission

Patient group	Event / N (%)		aRR (95% CI)
	AI-SaMD group, no. /N (%)	Standard-care group, no. /N (%)	
Death following CPR among patients with full-code status at admission	109 / 92,566 (0.12%)	84 / 69,898 (0.12%)	0.94 (0.76–1.15)

Adjusted rate ratios (aRRs) and 95% CIs were estimated using Poisson generalized linear mixed models with a hospital-level random intercept. The model was adjusted for sex, age, Charlson Comorbidity Index, hypertension, SOFA score at admission, season of admission, and department type.
Abbreviations: aRR, adjusted rate ratio; CI, confidence interval; CPR, cardiopulmonary resuscitation; SOFA, Sequential Organ Failure Assessment.

Supplementary Table S4. Per-Component Missing Rate of Baseline SOFA Score Under Two Laboratory Inclusion Windows

SOFA component	Sensitivity definition (post-admission only) (n = 164,761)	Primary definition (pre-admission 30-day window included) (n = 164,761)
Respiration (SpO_2)	7,346 (4.46%)	7,346 (4.46%)
Coagulation (platelet)	19,691 (11.95%)	13,254 (8.04%)
Liver (bilirubin)	27,523 (16.70%)	17,089 (10.37%)
Renal (creatinine)	22,910 (13.90%)	13,822 (8.39%)
Cardiovascular (SBP)	2,449 (1.49%)	2,449 (1.49%)
CNS (AVPU)	54,695 (33.20%)	44,445 (26.98%)

Abbreviations: SOFA, Sequential Organ Failure Assessment; SpO_2, peripheral oxygen saturation; SBP, systolic blood pressure; CNS, central nervous system; AVPU, Alert–Voice–Pain–Unresponsive

Supplementary Table S5. Propensity Score Matched Analysis (Exact-Matched on Hospital and Calendar Year) of Ward IHCA and In-Hospital Mortality with E-value Sensitivity Analysis

Outcome	AI-SaMD group<br><br>n = 11,832	Standard-care group<br><br>n = 11,832	Adjusted RR (95% CI)	P value	E-value (lower CI)
Ward IHCA, no. (%)	19 (0.16%)	26 (0.22%)	0.73 (0.40–1.32)	0.299	2.08 (1.00)
In-hospital mortality, no. (%)	132 (1.12%)	186 (1.57%)	0.71 (0.57–0.89)	0.003	2.17 (1.51)

Adjusted rate ratios were estimated by Poisson regression on a 1:1 propensity-score-matched sample (caliper, 0.2 SD of the logit; exact matching on hospital and calendar year); see Methods for full details. E-values are reported as point estimate (lower confidence limit).

Abbreviations: aRR, adjusted rate ratio; CI, confidence interval; IHCA, in-hospital cardiac arrest.

Supplementary Table S6. Association Between AI-SaMD Implementation and Clinical Outcomes

	AI-SaMD group	Standard-care group	Adjusted Risk Difference, pp (95% CI)	P value
**All included**	**n = 93,828**	**n = 70,933**		
Ward IHCA, no. (%)	108 (0.12%)	102 (0.14%)	-0.04 (-0.08, -0.01)	0.019
In-hospital mortality, no. (%)	983 (1.05%)	897 (1.26%)	-0.28 (-0.51, -0.05)	0.018
**Sepsis cohort**	**n = 5,269**	**n = 3,065**		
Ward IHCA, no. (%)	56 (1.06%)	51 (1.66%)	-0.66 (-1.20, -0.11)	0.018
In-hospital mortality, no. (%)	351 (6.66%)	301 (9.82%)	-3.71 (-6.16, -1.23)	0.003

Ward IHCA and in-hospital mortality are shown as count (%) per group. Adjusted risk differences (aRDs), expressed in percentage points (pp), with 95% confidence intervals were obtained by marginal standardization (g-computation) from Poisson generalized linear mixed-effects models with a log link and a hospital-level random intercept. All models were adjusted for sex, age, Charlson Comorbidity Index, hypertension, SOFA score at admission, season of admission, and department type. The sepsis cohort comprises admissions meeting the CDC Adult Sepsis Event surveillance criteria.

Abbreviations: aRD, adjusted risk difference; CDC, Centers for Disease Control and Prevention; CI, confidence interval; IHCA, in-hospital cardiac arrest; SOFA, Sequential Organ Failure Assessment.
